# Increase in dicentric chromosome formation after a single CT scan in adults

**DOI:** 10.1038/srep13882

**Published:** 2015-09-09

**Authors:** Yu Abe, Tomisato Miura, Mitsuaki A. Yoshida, Risa Ujiie, Yumiko Kurosu, Nagisa Kato, Atsushi Katafuchi, Naohiro Tsuyama, Takashi Ohba, Tomoko Inamasu, Fumio Shishido, Hideyoshi Noji, Kazuei Ogawa, Hiroshi Yokouchi, Kenya Kanazawa, Takashi Ishida, Satoshi Muto, Jun Ohsugi, Hiroyuki Suzuki, Tetsuo Ishikawa, Kenji Kamiya, Akira Sakai

**Affiliations:** 1 Dept. of Radiation Life Sciences, Fukushima Medical University School of Medicine, Fukushima, Japan; 2 Dept. of Pathologic Analysis, Hirosaki University Graduate School of Health Sciences, Hirosaki, Japan; 3 Dept. of Radiation Biology, Institute of Radiation Emergency Medicine, Hirosaki University, Hirosaki, Japan; 4 Dept. of Radiation Health Management, Fukushima Medical University School of Medicine, Fukushima, Japan; 5 Dept. of Radiology, Fukushima Medical University School of Medicine, Fukushima, Japan; 6 Dept. of Cardiology & Hematology, Fukushima Medical University School of Medicine, Fukushima, Japan; 7 Dept. of Pulmonary Medicine, Fukushima Medical University School of Medicine, Fukushima, Japan; 8 Dept. of Regenerative Surgery, Fukushima Medical University School of Medicine, Fukushima, Japan; 9 Dept. of Radiation Physics and Chemistry, Fukushima Medical University School of Medicine, Fukushima, Japan; 10 Dept. of Experimental Oncology, Research Institute for Radiation Biology and Medicine, Hiroshima University, Hiroshima, Japan; 11 Radiation Medical Science Center for the Fukushima Health Management Survey, Fukushima Medical University School of Medicine, Fukushima, Japan

## Abstract

Excess risk of leukemia and brain tumors after CT scans in children has been reported. We performed dicentric chromosome assay (DCAs) before and after CT scan to assess effects of low-dose ionizing radiation on chromosomes. Peripheral blood (PB) lymphocytes were collected from 10 patients before and after a CT scan. DCA was performed by analyzing either 1,000 or 2,000 metaphases using both Giemsa staining and centromere-fluorescence *in situ* hybridization (Centromere-FISH). The increment of DIC formation was compared with effective radiation dose calculated using the computational dosimetry system, WAZA-ARI and dose length product (DLP) in a CT scan. Dicentric chromosome (DIC) formation increased significantly after a single CT scan, and increased DIC formation was found in all patients. A good correlation between the increment of DIC formation determined by analysis of 2,000 metaphases using Giemsa staining and those by 2,000 metaphases using Centromere-FISH was observed. However, no correlation was observed between the increment of DIC formation and the effective radiation dose. Therefore, these results suggest that chromosome cleavage may be induced by one CT scan, and we recommend 2,000 or more metaphases be analyzed in Giemsa staining or Centromere-FISH for DCAs in cases of low-dose radiation exposure.

An epidemiologic investigation following atomic bomb (A-bomb) survivors in Hiroshima and Nagasaki revealed no significant health effects from radiation doses of 100 mSv or less^1^. Furthermore, studies analyzing the influence of chronic low-dose radiation exposure on inhabitants in high background radiation area (HBRA) revealed no increase in cancer and non-cancer mortality^2^,^3^. However, recent studies^4^,^5^ assessing cancer risk in children and adolescents following exposure to low-dose ionizing radiation from diagnostic computed tomography (CT) scans involving a cumulative dose of around 50 mSv indicated that chromosome aberrations may be induced by CT scanning. These chromosome aberrations that may cause cancer and hematological malignancies include chromosomal translocations, deletions, and inversions, resulting from the cleavage of chromosomes^6^,^7^,^8^. The dicentric chromosome (DIC) assay (DCA), which analyzes the number of DICs formed in peripheral blood (PB) lymphocytes provides an index of chromosome cleavage^9^. The critical variable is the lower limit of radiation dose for which an effect can be detected by DCA^9^,^10^. Conventional DCA involving the scoring of 1,000 metaphases is supposed to provide a lower limit of approximately 100 mGy of not just gamma, but also X-rays^11^. However, there are some reports of DCAs conducted in patients who received a CT scan^12^,^13^ in which the radiation dose was less 100 mSv. Although the detection of chromosomal aberrations using Giemsa staining is a conventional method for DCA, it however, requires a well-trained and skilled observer. Therefore the incorporation of easier performing fluorescence *in situ* hybridization (FISH) is expected to further enhance the value of DCA^14^. Giemsa staining is a conventional method for DCA, however, we are expecting that Centromere-FISH will replace Giemsa staining because it is easier to perform.

Here, we performed DCAs using both Giemsa staining and Centromere-FISH in adult PB lymphocytes after one CT scan to analyze the extent to which CT scan induces chromosomal breakage, which means cleavage of double-stranded DNA. At the same time, we confirmed that the dose of radiation in each CT scan was less than 100 mSv using the computational dosimetry system, WAZA-ARI^15^,^16^,^17^. Consequently, we detected DIC formation after one CT scan and a good correlation of DIC formation according to both methods by 2,000 or more metaphases analyses. Although the sample size is small, the finding of an increase in DIC formation after a single CT scan in the analysis of all 10 of 10 samples prompted us to make an early report.

## Results

### Subject background data

Background data pertaining to 10 patients are shown in Table 1. For patients with malignant lymphoma (ML) followed-up after chemotherapy and/or radiotherapy, at least 5 years had elapsed between those treatments and the study, and patients with a history of smoking had ceased smoking more than 10 years prior to the study. All patients had undergone chest X-ray during annual medical examinations. In addition, all patients except one (patient 10) underwent CT scans more than 5 times during the previous 5 years, and 3 ML patients underwent a positron emission tomography (PET) examination before this study. With respect to medication, one patient (patient 2) was given 2.5 mg of predonine every other day for hay fever. Three patients (patient 3, 4, and 8) were prescribed medication for hypertension, and one patient (patient 10) was prescribed medication for diabetes. It should be emphasized that two observers who evaluated the data were not informed of the patients’ backgrounds, and smoking history was deemed not to have an influence on DIC formation because 10 years had passed since smoking cessation in those patients.

### Analysis of DIC formation before and after CT scanning and the relationship between the increment of DIC formation and the dose of radiation exposure

**Figure 1:**
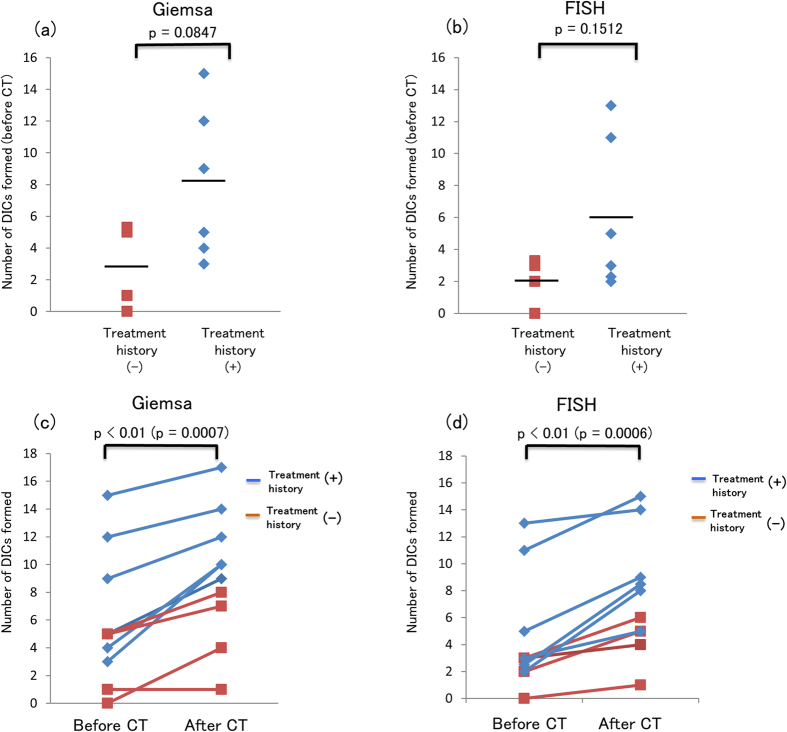
Number of DICs formed before the CT scan and comparison of the number of DICs formed before and after the CT scan. The line indicates the mean value. There was no significant difference between patients with and without treatment history, as determined using both Giemsa staining (p = 0.0847) (**a**) and Centromere-FISH (p = 0.1512) (**b**). Significantly more DICs were formed after the CT scan than before the CT scan, as determined using both Giemsa staining (p = 0.0007) (**c**) and Centromere-FISH (p = 0.0006) (**d**).

A total of 2,000 metaphases were analyzed for DIC formation using Giemsa staining and Centromere-FISH before and after CT scan. The number of DICs formed and their distribution in cells are shown in Table S1. The radiation dose estimated based on the increment of DIC formation as determined by Giemsa staining and Centromere-FISH in comparison with the standard dose-response curve and the effective radiation dose of each CT scan as calculated using WAZA-ARI and DLP are shown in Table 2. Because the increment of DIC formation in patient 4, 7, and 8 in analyses using Giemsa staining and in patient 2, 4, 8, and 10 in analyses using Centromere-FISH were lower than the number of DICs formed in the background of the standard dose-response curve, the estimated dose in those patients were not available. The number of DICs formed before the CT scan was compared between patients with and without previous chemotherapy and/or radiotherapy (Fig. 1a,b). The number of DICs formed tended to be higher (but not significantly) in patients with a treatment history compared with patients without a history as determined using both methods. The number of DICs formed after the CT scan was significantly higher than the number formed prior to the scan, as determined with both Giemsa staining (*p* < 0.01) (Fig. 1c) and Centromere-FISH (*p* < 0.01) (Fig. 1d).

**Figure 2:**
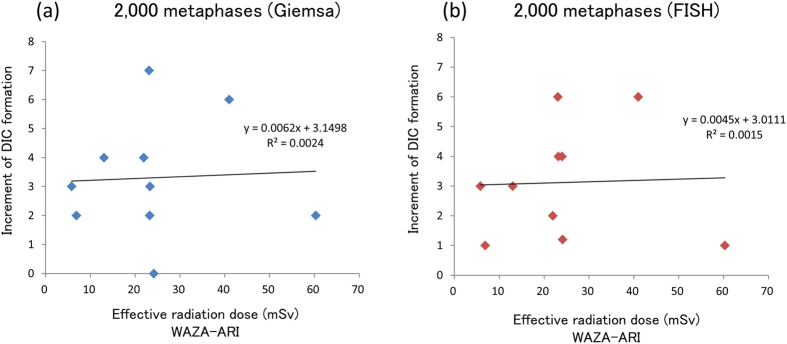
Relationship between the increment of DIC formation and the effective radiation dose, as calculated using WAZA-ARI. No correlation was observed with the results of either Giemsa staining (R^2^ = 0.00238) (**a**) or Centromere-FISH (R^2^ = 0.00147) (**b**).

We then analyzed the relationship between the increment of DIC formation after CT scanning and the effective radiation dose as calculated using WAZA-ARI or DLP. No significant correlation was observed using either method (Fig. 2 and Fig. S1). A good correlation was observed between the effective radiation dose as calculated using WAZA-ARI and DLP (Fig. S2). It must be noted, however, that DIC formation could be detected after one CT scan, in which the effective radiation dose was less than 70 mSv.

### Comparison of DIC analyses of 1,000 and 2,000 metaphases and/or using Giemsa staining and Centromere-FISH

**Figure 3:**
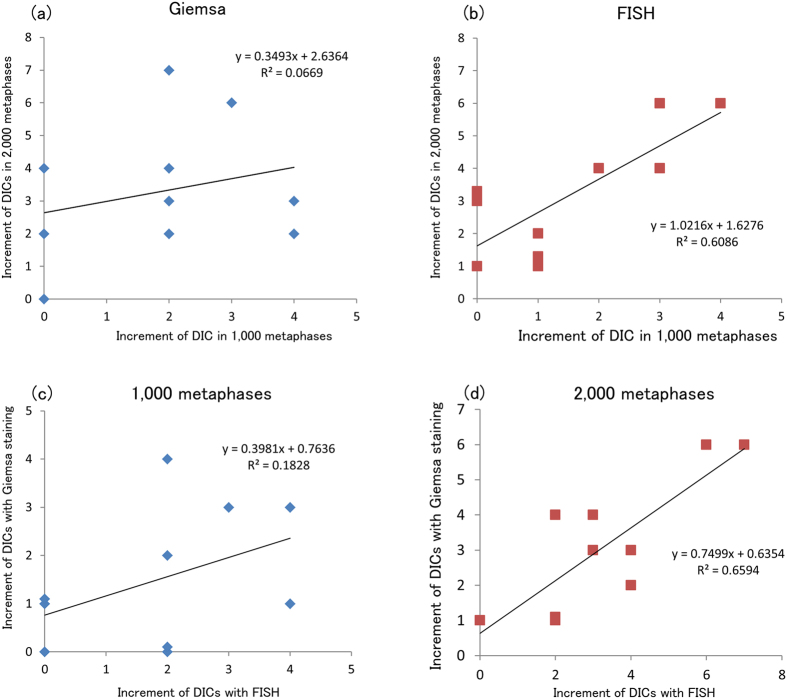
Relationship of the increment of DIC formation between analysis of 1,000 and 2,000 metaphases using either Giemsa staining or Centromere-FISH, or between the results of Giemsa staining and Centromere-FISH in analysis of either 1,000 or 2,000 metaphases. No correlation was observed with the results of Giemsa staining (R^2^ = 0.06692) (**a**), but a correlation was observed with the results of Centromere-FISH (R^2^ = 0.60864) (**b**). No correlation was observed in the analysis of 1,000 metaphases (R^2^ = 0.18283) (**c**), but a correlation was observed in the analysis of 2,000 metaphases (R^2^ = 0.65942) (**d**).

Although the International Atomic Energy Agency (IAEA) recommends that 1,000 cells be analyzed for biodosimetry using DCA at the time of a radiation exposure emergency^12^, the number of cells that must be analyzed to detect DIC formation following exposure to low doses radiation (less than 100 mSv) must be increased to reduce the statistical uncertainty. Here, we analyzed 2,000 metaphases in a patient and then assessed the reliability of analysis of 1,000 and 2,000 metaphases using both methods. The number of DICs formed before and after the CT scan as determined using Giemsa staining and Centromere-FISH is shown in Table S1 and Table S2. We found a good correlation between the increment of DIC formation determined by analysis of 1,000 and those by 2,000 metaphases using Centromere-FISH (Fig. 3b) but not Giemsa staining (Fig. 3a). Furthermore, we found a good correlation between the increment of DIC formation using Giemsa staining and those using Centromere-FISH in the analysis of 2,000 metaphases (Fig. 3d) but not in the analysis of 1,000 metaphases (Fig. 3c). Therefore, it is recommended that 2,000 metaphases or more be analyzed by Giemsa staining and 1,000 metaphases be analyzed by Centromere-FISH to achieve an equivalent level precision.

## Discussion

In this study, we performed DCAs in adult PB lymphocytes to determine whether chromosome aberrations occur in response to low-dose radiation exposure (i.e., less than 100 mSv). The dose of radiation exposure resulting from one CT scan is around 10 mSv, and the dose increases to around 50 mSv with a CT scan of the whole body. Therefore, the use of DCA in patients undergoing CT scanning is thought to be appropriate, as the dose of radiation is low. We expected to find an increase in DIC formation following CT scanning. The DCA is the gold standard method of biological dosimetry^9^,^10^, and can be used to evaluate the dose of whole-body radiation exposure in a radiation disaster by comparing the number of DICs formed in lymphocytes to the standard dose-response curve. With respect to radiation exposure resulting from CT scanning, only lymphocytes localized in the tissues being scanned are exposed to the ionizing radiation, and they are diluted by the non-irradiated cells in the blood circulation afterwards, especially, when a small volume is irradiated by CT scanning, and distributed equally throughout the body. Therefore, it is believed that the number of DICs formed in PB lymphocytes is lower than the true number induced by the exposure to ionizing radiation^10^,^18^. Löbrich *et al*. reported that the number of DNA double-strand breaks (DSBs) induced by CT scan showed linear dose response curve^19^. However, their method was the enumerating γ-H2AX foci as a measure for DSBs in lymphocytes at 30 min and 60 min after a single examination of CT scan. This method would be reasonable if we could perform it within a few hours because number of γ-H2AX foci reduces quickly due to DSB repair. On the other hand, analysis of the number of DICs formed includes factors of abnormal chromosomal repair in addition to DSBs. Because lymphocytes with DICs usually die within several months, we collected blood within 3–28 days of each CT scan. It is unusual to detect a significant decrease in the number of DICs formed by 2,000 metaphases analysis, especially in low-dose radiation exposure like CT scan, as some lymphocytes with DIC just entering into M-phase will die within one month. In addition, some chromosome cleavages resulting from ionizing radiation may be repaired. Considering these factors, it is notable that we found an increase in DIC formation in all 10 patients after only one CT scan.

The analysis of 1,000 metaphases by DCA is recommended in cases of exposure to γ-ray radiation doses around 100 mSv^12^,^10^, and this can be reduced to approximately 70 mSv by analyzing around 10,000 metaphases^10^. Here, we analyzed 2,000 metaphases because it was impossible to analyze 10,000. Suto *et al*. performed DCAs of 1,000 metaphases using Giemsa staining of the PB lymphocytes of nuclear workers who engaged in emergency response tasks at the Fukushima Daiichi Nuclear Power Station and compared their results with exposure recorded using personal physical dosimeters. They found a correlation between the DCA results and the personal dosimeter records with respect to radiation doses in the range of 26–171 mSv^20^. Iwasaki *et al*. demonstrated a linear dose response with respect to chromosome aberrations in human lymphocytes exposed to less than 50 mSv of γ-rays in an analysis of more than 5,000 metaphases using a semi-automated metaphase-finding/relocation system^21^. These studies confirm that the DCA is useful for detecting exposure to radiation doses less than 100 mSv, although the analysis of more than 2,000 metaphases may be necessary. As Shi *et al*. previously reported that FISH is more accurate than conventional Giemsa staining for dose estimation, especially in samples exposed to high doses^11^, we also recommend the use of FISH for DCA in cases of low-dose radiation exposure because in contrast to Giemsa staining, we found a good correlation between the results of analyses of 1,000 and 2,000 metaphases using Centromere-FISH in this study.

The increase in the number of DICs formed as observed using both Giemsa staining and Centromere-FISH following CT scanning suggests that radiation doses less than 100 mSv are sufficient to cleave double-stranded DNA. Chemotherapy and radiotherapy induce DNA damage and cause chromosomal instability^22^,^23^. However, DICs are mitotically unstable and are gradually eliminated from the body because, unlike translocated chromosomes, they are unable to pass through repeated cell divisions^12^. We don’t think that prior treatment caused persistent DIC formation because more than 5 years had passed since any of the patients enrolled in this study had undergone chemotherapy or radiotherapy treatment. Still, the number of DICs formed tended to be higher in those patients with a history of treatment. We hypothesize that this is due to those patients having undergone regular CT scans to evaluate treatment progress.

Lymphocytes with DICs are unstable and do not live more than several months, however, reports suggest that the number of DICs formed increases over the lifetime of the persons living in HBRAs^24^,^25^. Therefore, the number of DICs formed increases in relation to accumulated dose of radiation exposure, suggesting that, following chronic low-dose radiation exposure, lymphocytes with DICs might accumulate rather than die, or lymphocytes with DICs simply might accumulate by chromosomal instability due to aging. Based on previous studies^26^,^27^, which reported that dicentric chromosomes and chromosome translocations are produced in about an equal ratio, we speculate that there may also be an increase in chromosomal translocation, which plays an important role in carcinogenesis, in HBRAs. However, an increase in the number of cancer patients in HBRAs has not been observed^1^,^2^.

The dose of radiation exposure associated with a CT scan is relatively high for medical radiation-related exposure, and the area of the body surface exposed to ionizing radiation is large in intensive whole-body CT scans for cancer, thus presenting a different exposure scenario compared with local radiotherapy involving high dose of radiation. The accumulation of radiation doses is a particularly critical problem in patients undergoing frequent CT scans during medical treatment. Excess risks of leukemia and brain tumors in children after CT scans were recently reported^4^,^5^; however, no analyses of chromosome aberrations were performed in those studies. Although it is thought that children and adults have differing sensitivity to ionizing radiation, we found that the DICs formed in the PB lymphocytes of adults after exposure to 5–60 mSv of radiation associated with a single CT scan.

The fact that chemotherapy and radiotherapy can induce a second cancer is well known. However, the cause of such cancer is not considered due to chromosomal translocations induced by high-dose radiation exposure but rather to somatic mutations induced by low-dose radiation exposure. Therefore, it cannot be said that the DICs formed after a CT scan will immediately lead to the occurrence of diseases such as cancers. However, DICs are formed as a result of cleavage of double-stranded DNA, suggesting that chromosome aberrations such as translocations or deletions may occur after a CT scan. In a future study, we plan to perform chromosome painting analyses whether CT scanning induces chromosome translocations.

## Patients and Methods

### Ethics Statement

The samples and the medical records used in our study have been approved by the Ethics Committee of the Fukushima Medical University School of Medicine (approval number 1577). Written informed consent was obtained from all participants for analysis of PB samples, and the methods were carried out in accordance with approved guidelines of the Council for International Organizations of Medical Science^28^.

### Subjects

The study involved 10 patients (3 males and 7 females) aged 62–81 years (mean 68 years) who had medical examinations in hematologic internal medicine, respiratory internal medicine, and respiratory surgery at Fukushima Medical University Hospital. Data regarding past history of disease and treatment, CT scans, and smoking status for the subjects are shown in Table 1. Patients who received previous radiotherapy or chemotherapy did not take any treatment within 1 year before entry into this study.

### Separation of lymphocytes from PBs and cell culture conditions

Heparinized PBs from each patient before and after (within 3–28 days) the CT scan, and mononuclear blood cells were isolated using BD Vacutainer CPT tubes (BD Biosciences, San Jose, CA, USA) according to the manufacturer’s instructions. Cells were suspended in RPMI 1640 medium (Nacalai Tesque, Kyoto, Japan) containing 20% fetal bovine serum (Equitech Bio, Keilor East, Australia), 2% phytohaemagglutinin-HA15 (Remel, Lenexa, KS, USA), and 60 μg/ml of kanamycin solution (Life Technologies, Carlsbad, CA, USA) in a 15-ml Falcon tube. Lymphocytes were cultured in a 5% humidified CO_2_ incubator at 37 °C for 48 h. First-division metaphases were obtained by treatment with colcemid (final concentration, 0.05 μg/ml; Life Technologies) for 48 h.

### Cell harvesting

After 48 h of culture, cells were harvested, treated with 0.075 M KCL, and fixed with methanol/acetic acid (3:1) according to the standard cytogenetic procedure^9^,^12^. Finally, the cell pellets were suspended in 1–2 ml of fixative, depending on the size of the pellets. One drop (around 20 μl) of the suspension was dispensed onto a slide and spread on water bath.

### Giemsa staining

Each slide was immersed in 5% Giemsa (Merck Millipore, Darmstadt, Germany) solution for 15 min, and then washed with distilled water and air dried.

### Centromere-fluorescence *in situ* hybridization (Centromere-FISH)

First, each slide was dried at 65 °C for 1 h or more for hardening. Next, 5–6 μl of Poseidon probe (KRATECH, Amsterdam, The Netherlands) solution was applied per 22 × 22-mm area and covered with a glass coverslip and sealed with paper bond. Nuclear DNA was denatured by incubating the slides on a hot plate at 72 °C for 4 min and then the slides were incubated overnight at 37 °C in a humidified chamber to allow for hybridization. The glass coverslips were removed and the slides were washed in post-wash buffers I (0.4 × SSC/0.3% Triton X-100) at 72 °C for 2 min and then washed in wash buffers II (2 × SSC/0.1% Triton X-100) at RT for 1 min. Subsequently, the slides were dehydrated by successive in 70% and 100% ethanol for 5 min and then air dried at RT. Finally, nuclei were counterstained with Vectashield Mounting Medium with DAPI (Vector, Burlingame, USA), covered with a glass coverslip, and sealed with nail polish.

### Image capturing and chromosome analysis

Soon after completing chromosome preparations, Giemsa-stained metaphase spreads and Centromere-FISH images were captured in the AutoCapt mode using two sets of AXIO Imager Z2 microscopes (Carl Zeiss AG, Oberkochen, Germany) equipped with CCD cameras and Metafer 4 software (MetaSystems GmbH, Altlussheim, Germany), respectively. Metaphases for scoring were selected in manual mode. Chromosome analysis was performed according to the IAEA manual (IAEA 2001)^9^ by two trained, experienced observers. Using the selected metaphase images, all observable aberrations in 2,000 or more metaphases were scored and classed as dicenrics or multicentrometrics (chromosomes with three or more centromeres). Other chromosome- or chromatid-type aberrations, such as rings, acentric fragments, and chromatid exchanges, were also recorded. Metaphases with less than 45 centromeres were omitted from analysis.

### Calculation of effective CT scan radiation dose and dose length product (DLP)

A Toshiba Aquilion model 64 CT scanner was used in this study, with a tube voltage of 120 kV. The effective radiation dose was calculated by inputting data regarding age, sex, and initiation and the end position of CT scan into the computational dosimetry system (WAZA-ARI: http://waza-ari.nirs.go.jp/waza_ari/login/)^15^,^16^,^17^. DLP was calculated according to CT dose index (CTDI), which is set uniformly in the CT scanner, and length (L) of axial CT scanning range of a body as below:
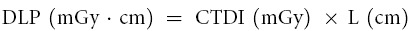


### Statistical analysis

The Student’s *t*-test was used to compare the numbers of DICs formed before CT scanning in patients with or without the previous treatment. The Student’s paired *t*-test was used to compare the numbers of DICs formed before and after CT scanning. The relationship between the increments of DIC formation and the effective radiation doses calculated by WAZA-ARI, the relationship between the increments of DIC formation and DLP, the relationship between the increments of DIC formation determined by analysis of 1,000 and those determined by analysis of 2,000 metaphases, the relationship between the increment of DIC formation using Giemsa staining and Centromere-FISH, and the relationship between the effective radiation doses calculated by WAZA-ARI and DLP were evaluated using simple linear regression analysis. Analyses were performed using STATA software, Version 11.1 (StataCorp, College Station, TX, USA). *P* values of less than 0.05 were regarded as statistically significant.

## Additional Information

**How to cite this article**: Abe, Y. *et al*. Increase in dicentric chromosome formation after a single CT scan in adults. *Sci. Rep*. **5**, 13882; doi: 10.1038/srep13882 (2015).

## Supplementary Material

Supplementary Information

## Figures and Tables

**Table 1:** Patient background data.

**Patient No.**	**Disease**	**Part of body examined in CT scan**	**Days from CT scan to PB collection**	**Treatment**#1	**Smoking status**	**Past CT examination**#3	**Other X-ray examinations**#4
1	Lung cancer	Chest	8	Operation	(−)	(+)	Chest, UGI, PET
2	Lymphoma	Cervix, Chest, Abdomen, Pelvis	3	Chemotherapy	(−)	(+)	Chest, UGI
3	Lymphoma	Chest, Abdomen, Pelvis	11	Chemotherapy & Radiotherapy	(−)	(+)	Chest, UGI, PET
4	Chest abnormal shadow	Chest	15	(−)	(−)	(+)	Chest, UGI
5	Chest abnormal shadow	Chest	22	(−)	(+)#2	(+)	Chest, UGI
6	Lymphoma	Chest, Abdomen, Pelvis	14	Chemotherapy	(−)	(+)	Chest, UGI
7	Lymphoma	Cervix, Chest, Abdomen, Pelvis	2	Chemotherapy & Radiotherapy	(+)#2	(+)	Chest, UGI, PET
8	Lymphoma	Cervix, Chest, Abdomen, Pelvis	28	Chemotherapy	(−)	(+)	Chest, UGI
9	Lymphoma	Chest, Abdomen, Pelvis	7	Chemotherapy	(+)#2	(+)	Chest, UGI, PET
10	Chest abnormal shadow	Chest	14	(−)	(−)	(+)	Chest, UGI

^#1^Chemotherapy or radiotherapy had been performed at least five years before this study.

^#2^These patients had given up smoking at least ten years before this study.

^#3^All patients except one (No. 10) took examinations of CT scanning more than 5 times during the past 5 years.

^#4^UGI: X-ray examination of the upper gastrointestinal tract, PET: positron emission tomography.

**Table 2:** Increment in DIC formation resulting from CT scan.

**Patient No.**	**Number of DICs/metaphase**		**Increment**	**WAZA-ARI (mSv)**	**DLP (mGy•cm)**
	**Before CT**	**After CT**			
**(a) Giemsa staining, 2,000 metaphases**					
1	5	8	3/2000	5.78	619.1
2	5	9	4/2000	21.90	2557.8
3	9	12	3/2000	23.26	2514
4	5	7	2/2000	6.85	1369.7
5	0	4	4/2000	12.99	1880.3
6	3	10	7/2000	23.07	3265.6
7	15	17	2/2000	23.21	5321.6
8	12	14	2/2000	60.27	5501.3
9	4	10	6/2000	40.96	2788.6
10	1	1	0/2000	24.13	1393.4
**(b) Centromere-FISH, 2,000 metaphases**					
1	2	5	3/2000	5.78	619.1
2	3	5	2/2000	21.90	2557.8
3	5	9	4/2000	23.26	2514
4	3	4	1/2000	6.85	1369.7
5	3	6	3/2000	12.99	1880.3
6	2	8	6/2000	23.07'	3265.6
7	11	15	4/2000	23.21	5321.6
8	13	14	1/2000	60.27	5501.3
9	2	8	6/2000	40.96	2788.6
10	0	1	1/2000	24.13	1393.4
