# Supplementary material for: Increase in dicentric chromosome formation after a single CT scan in adults
Source: Sci Rep. 2015 Sep 9;5:13882. doi: 10.1038/srep13882 (PMC4563376; doi:10.1038/srep13882)
Supplement: Supplementary Information [file srep13882-s1.pdf]

[Supplementary Information]

Increase in dicentric chromosome formation after a single CT scan in adults

Yu Abe[1], Tomisato Miura[2], Mitsuaki A Yoshida[3], Risa Ujiie[1], Yumiko Kurosu[1], Nagisa Kato[1], Atsushi Katafuchi[1], Naohiro Tsuyama[1], Takashi Ohba[4], Tomoko Inamasu[4], Fumio Shishido[5], Hideyoshi Noji[6], Kazuei Ogawa[6], Hiroshi Yokouchi[7], Kenya Kanazawa[7], Takashi Ishida[7], Satoshi Muto[8], Jun Ohsugi[8], Hiroyuki Suzuki[8], Tetsuo Ishikawa[9, 11], Kenji Kamiya[10, 11], Akira Sakai[1, 11*]

[1]Dept. of Radiation Life Sciences, Fukushima Medical University School of Medicine, Fukushima, Japan
[2]Dept. of Pathologic Analysis, Hirosaki University Graduate School of Health Sciences, Hirosaki, Japan
[3]Dept. of Radiation Biology, Institute of Radiation Emergency Medicine, Hirosaki University, Hirosaki, Japan
[4]Dept. of Radiation Health Management, Fukushima Medical University School of Medicine, Fukushima, Japan
[5]Dept. of Radiology, Fukushima Medical University School of Medicine, Fukushima, Japan
[6]Dept. of Cardiology & Hematology, Fukushima Medical University School of Medicine, Fukushima, Japan
[7]Dept. of Pulmonary Medicine, Fukushima Medical University School of Medicine, Fukushima, Japan
[8]Dept. of Regenerative Surgery, Fukushima Medical University School of Medicine, Fukushima, Japan
[9]Dept. of Radiation Physics and Chemistry, Fukushima Medical University School of Medicine, Fukushima, Japan
[10]Dept. of Experimental Oncology, Research Institute for Radiation Biology and Medicine, Hiroshima University, Hiroshima, Japan
[11]Radiation Medical Science Center for the Fukushima Health Management Survey, Fukushima Medical University School of Medicine, Fukushima, Japan

*Correspondence:
Akira Sakai, M.D., Ph.D.
Dept. of Radiation Life Sciences
Fukushima Medical University School of Medicine
1 Hikarigaoka, Fukushima, 960-1295 Japan
TEL: +81-24-547-1703
FAX: +81-24-547-1704
E-mail: sakira@fmu.ac.jp

Table S1a. Analysis of DIC formation before and after the CT scan (Giemsa staining, 2,000 metaphases)

Patient No.	Timing	Number of cells scored	Number of DICs formed	Number of cells with aberrations#	DICs/cell	Increment of DIC formation	DIC distribution					
							0	1	2	3	4	≥5
1	Before CT	2000	5	20	0.003	3 / 2000	1995	5	0	0	0	0
	After CT	2000	8	20	0.004		1993	6	1	0	0	0
2	Before CT	2000	5	27	0.003	4 / 2000	1995	5	0	0	0	0
	After CT	2000	9	25	0.005		1991	9	0	0	1 rogue cell	0
3	Before CT	2000	9	38	0.005	3 / 2000	1991	9	0	0	0	2 rogue cells
	After CT	2000	12	22	0.006		1989	10	1	0	1 rogue cell	0
4	Before CT	2000	5	17	0.003	2 / 2000	1995	5	0	0	0	0
	After CT	2000	7	18	0.004		1993	7	0	0	0	0
5	Before CT	2000	0	6	0.000	4 / 2000	2000	0	0	0	0	0
	After CT	2000	4	24	0.002		1996	4	0	0	0	0
6	Before CT	2000	3	17	0.002	7 / 2000	1997	3	0	0	0	0
	After CT	2000	10	32	0.005		1990	10	0	0	0	0
7	Before CT	2000	15	37	0.008	2 / 2000	1985	15	0	0	1 rogue cell	0
	After CT	2000	17	51	0.009		1983	17	0	0	0	0
8	Before CT	2000	12	56	0.006	2 / 2000	1988	12	0	0	0	0
	After CT	2000	14	56	0.007		1986	14	0	0	0	0
9	Before CT	2000	4	23	0.002	6 / 2000	1997	2	1	0	0	0
	After CT	2000	10	29	0.005		1990	10	0	0	0	0
10	Before CT	2000	1	7	0.001	0 / 2000	1999	1	0	0	0	0
	After CT	2000	1	7	0.001		1999	1	0	0	0	0

Chromosomal aberrations including dicentric, tricentric, centric ring, acentric ring, minute, and marker chromosome and fragment

Table S1b. Analysis of DIC formation before and after the CT scan (Centromere-FISH, 2,000 metaphases)

Patient No.	Timing	Number of cells scored	Number of DICs formed	Number of cells with aberrations[#]	DICs/cell	Increment of DIC formation	DIC distribution					
							0	1	2	3	4	≥5
1	Before CT	2000	2	15	0.001	3 / 2000	1998	2	0	0	0	0
	After CT	2000	5	27	0.003		1995	5	0	0	0	0
2	Before CT	2000	3	14	0.002	2 / 2000	1997	3	0	0	0	0
	After CT	2000	5	16	0.003		1995	5	0	0	0	0
3	Before CT	2000	5	24	0.003	4 / 2000	1995	5	0	0	0	0
	After CT	2000	9	36	0.005		1991	9	0	0	0	0
4	Before CT	2000	3	32	0.002	1 / 2000	1997	3	0	0	0	0
	After CT	2000	4	20	0.002		1996	4	0	0	0	0
5	Before CT	2000	3	26	0.002	3 / 2000	1997	3	0	0	0	0
	After CT	2000	6	24	0.003		1994	6	0	0	0	0
6	Before CT	2000	2	10	0.001	6 / 2000	1998	2	0	0	0	0
	After CT	2000	8	26	0.004		1992	8	0	0	0	0
7	Before CT	2000	11	29	0.006	4 / 2000	1989	11	0	0	0	0
	After CT	2000	15	42	0.008		1985	15	0	0	0	0
8	Before CT	2000	13	51	0.007	1 / 2000	1987	13	0	0	0	0
	After CT	2000	14	52	0.007		1986	14	0	0	0	0
9	Before CT	2000	2	12	0.001	6 / 2000	1998	2	0	0	0	0
	After CT	2000	8	25	0.004		1992	8	0	0	0	0
10	Before CT	2000	0	4	0.000	1 / 2000	2000	0	0	0	0	0
	After CT	2000	1	7	0.001		1999	1	0	0	0	0

Chromosomal aberrations including dicentric, tricentric, centric ring, acentric ring, minute, and marker chromosome and fragment

Table S2a. Analysis of DIC formation (Giemsa staining, 1,000 metaphases)

Patient No.	DICs formed/metaphase		Increment of DIC formation
	Before CT	After CT	
1	3	5	2/1000
2	5	5	0/1000
3	7	11	4/1000
4	4	4	0/1000
5	0	2	2/1000
6	2	4	2/1000
7	9	11	2/1000
8	5	9	4/1000
9	2	5	3/1000
10	1	0	-1/1000

Table S2b. Analysis of DIC formation (Centromere-FISH, 1,000 metaphases)

Patient No.	DICs formed/metaphase		Increment of DIC formation
	Before CT	After CT	
1	2	2	0/1000
2	1	2	1/1000
3	2	5	3/1000
4	2	2	0/1000
5	2	2	0/1000
6	1	5	4/1000
7	5	7	2/1000
8	7	8	1/1000
9	1	4	3/1000
10	0	1	1/1000

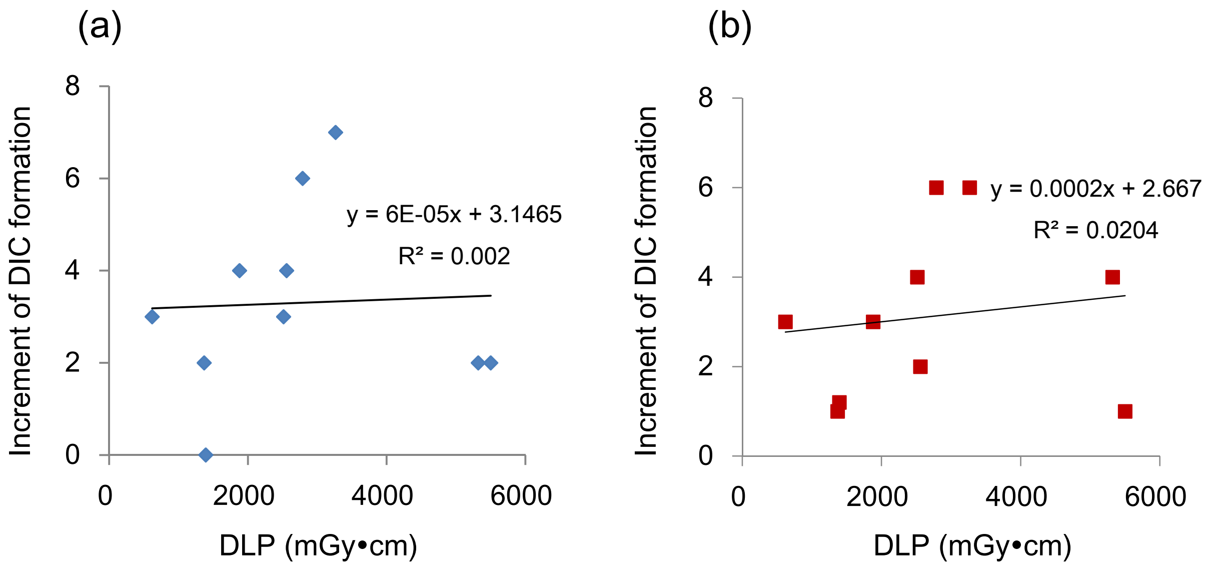
(a)
y = 6E-05x + 3.1465
R² = 0.002
Increment of DIC formation
DLP (mGy•cm)
0
2
4
6
8
0
2000
4000
6000
(b)
y = 0.0002x + 2.667
R² = 0.0204
Increment of DIC formation
DLP (mGy•cm)
0
2
4
6
8
0
2000
4000
6000

Figure S1.

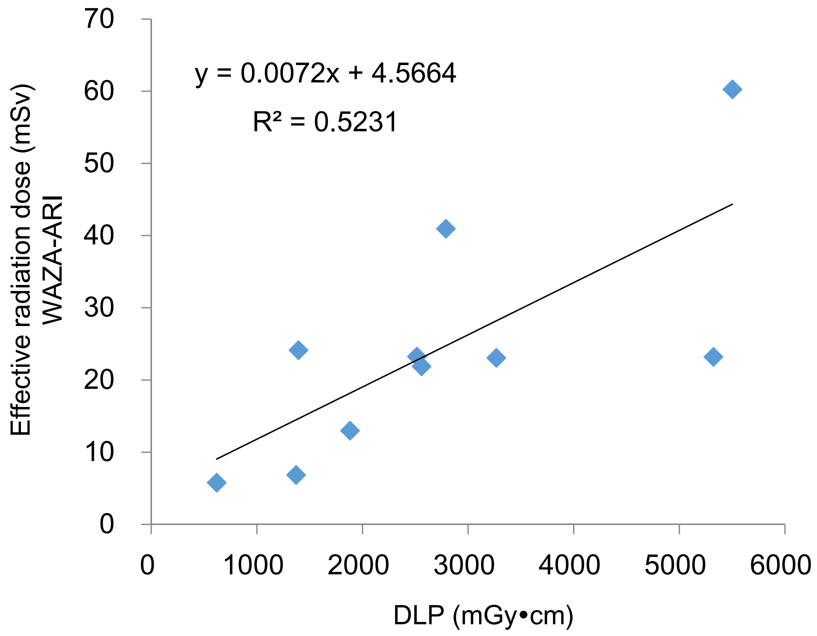
y = 0.0072x + 4.5664
R² = 0.5231
Effective radiation dose (mSv)
WAZA-ARI
DLP (mGy•cm)
0
10
20
30
40
50
60
70
0
1000
2000
3000
4000
5000
6000

Figure S2.

Supplementary Information

Table S1a. Analysis of DIC formation before and after the CT scan (Giemsa staining, 2,000 metaphases)

Table S1b. Analysis of DIC formation before and after the CT scan (Centromere-FISH, 2,000 metaphases)

Table S2a. Analysis of DIC formation (Giemsa staining, 1,000 metaphases)

Table S2b. Analysis of DIC formation (Centromere-FISH, 1,000 metaphases)

Figure S1. Relationship between the increment of DIC formation and DLP in analysis of 2,000 metaphases using either Giemsa staining or Centromere-FISH. No correlation was observed with the results of either Giemsa staining ($R^2 = 0.00197$) (a) or Centromere-FISH ($R^2 = 0.02036$) (b).

Figure S2. Relationship between DLP and the effective radiation dose, as calculated using WAZA-ARI. A correlation was observed between them ($R^2 = 0.52312$).
